# Aspirin Resistance in the Acute Stages of Acute Ischemic Stroke Is Associated with the Development of New Ischemic Lesions

**DOI:** 10.1371/journal.pone.0120743

**Published:** 2015-04-07

**Authors:** Joon-Tae Kim, Suk-Hee Heo, Ji Sung Lee, Min-Ji Choi, Kang-Ho Choi, Tai-Seung Nam, Seung-Han Lee, Man-Seok Park, Byeong C. Kim, Myeong-Kyu Kim, Ki-Hyun Cho

**Affiliations:** 1 Department of Neurology, Chonnam National University Hospital, Gwangju, Korea; 2 Department of Radiology, Chonnam National University Hwasun Hospital, Hwasun, Jeonnam, Korea; 3 Clinical Trial Center, Asan Medical Center, Seoul, Korea; St Michael's Hospital, University of Toronto, CANADA

## Abstract

**Background:**

Aspirin is a primary antiplatelet agent for the secondary prevention of ischemic stroke. However, if aspirin fails to inhibit platelet function, as is expected in acute ischemic stroke (AIS), it may increase the rate of early clinical events. Therefore, we sought to determine whether aspirin resistance in the acute stage was associated with early radiological events, including new ischemic lesions (NILs).

**Methods:**

This study was a single-center, prospective, observational study conducted between April 2012 and May 2013. Aspirin 300 mg was initially administered followed by maintenance doses of 100 mg daily. The acute aspirin reaction unit (aARU) was consistently measured after 3 hours of aspirin loading. An aARU value ≥550 IU was defined as biological aspirin resistance (BAR). NILs on follow-up diffusion-weighted imaging (DWI) were defined as lesions separate from index lesions, which were not detected on the initial DWI.

**Results:**

A total of 367 patients were analyzed in this study. BAR in aARU was detected in 60 patients (16.3%). On follow-up DWI, 81 patients (22.1%) had NILs, which were frequently in the same territory as the index lesions (79%), pial infarcts (61.7%), and located within the cortex (59.3%). BAR was independently associated with NILs on follow-up DWI (adjusted OR 2.00, 95% CIs 1.01–3.96; p = 0.047).

**Conclusion:**

In conclusion, BAR in aARU could be associated with NILs on follow-up DWI in AIS. Therefore, a further prospective study with a longer follow-up period is necessary to evaluate the clinical implications of aARU in AIS.

## Introduction

Aspirin is a primary antiplatelet agent used for the secondary prevention of ischemic stroke [1]. The relative stroke risk reduction associated with aspirin therapy has been estimated to be 15% [2]. However, approximately 10% to 20% of patients with coronary artery diseases treated with aspirin may experience a recurrent vascular event [3–5]. In addition, the effect of aspirin may not be uniform in individual patients. Aspirin responsiveness can vary in different patients according to physiological or pathological conditions that affect the drug’s pharmacokinetic or pharmacodynamic mechanisms, such as drug compliance and drug interactions [6].

Because the effects of aspirin begin promptly after aspirin loading, aspirin has been considered to be a first-line medication for preventing early recurrence in patients with acute ischemic stroke [7]. However, it is unclear whether aspirin limits the neurological consequences of acute ischemic stroke itself [7]. In addition, if aspirin fails to fully inhibit the production of platelet thromboxane A_2_ or to inhibit platelet function, as expected in patients with acute ischemic stroke [6, 8], it will increase the rate of early recurrence or new ischemic lesions (NIL) on follow-up imaging.

In several studies, aspirin resistance has been associated with more frequent early neurologic deterioration (END), less frequent early clinical improvement, and distant early recurrent ischemic lesions (ERIL) in patients with acute ischemic stroke [9–11]. These types of early events develop in approximately 12%–40% of patients with acute ischemic stroke and are associated with a worse clinical outcome [12–14]. However, because these studies were either retrospective in nature or small in size, their results should be interpreted cautiously, and larger prospective studies are required to confirm their results.

We hypothesized that the acute effects of aspirin may play an important role in preventing early ischemic events or radiologic changes on follow-up imaging in patients with acute ischemic stroke. If early clinical or radiological events in patients with acute ischemic stroke are associated with aspirin resistance, it may be helpful to assess aspirin responsiveness in these patients to prevent poor outcomes. Therefore, in this prospective study, we sought to determine whether aspirin responsiveness in the acute stage, as measured by the Rapid Platelet Function Assay, is associated with early radiologic changes, including NILs, in patients with acute ischemic stroke. In addition, we investigated which parameters of END may be associated with aspirin resistance in patients with acute ischemic stroke.

## Materials and Methods

### Patients

This work was a part of an ongoing single-center prospective observational study (S1 Text). Patients were consecutively recruited from the Cerebrovascular Center at Chonnam National University Hospital in Korea between April 2012 and May 2013. These patients (1) presented and were evaluated within the first 24 hours of symptom onset; but this criteria was changed into patients within 3 days of stroke onset since July 2012 in order to fast enrollments, (2) had positive lesions that were visualized using diffusion-weighted imaging (DWI), (3) had no potential risks of cardioembolism, and (4) provided written informed consent. The exclusion criteria included other etiologies from the Trial of Org 10172 in Acute Stroke Treatment (TOAST) classifications, thrombolysis, malignant infarction (lesion size >two-thirds of the middle cerebral artery territory and bilateral brainstem infarction, including occlusion of the top of the basilar artery), early loss to follow-up (within 5 days), chronic use of non-steroid anti-inflammatory drugs (>3 days per week during the most recent 3 months), a history of recent hemorrhagic disorders within the most recent 4 weeks, coagulopathies, thrombocytopenia (<90,000/μl), low hematocrit (<29%), and chronic liver or renal diseases.

### Ethics Statement

This study was approved by the Institutional Review Board (IRB) of Chonnam National University Hospital, and all of the clinical investigations of this study were conducted in accordance with the principles expressed in the Declaration of Helsinki. Written informed consent was obtained from either each participant or their families.

### Clinical evaluation

Demographic, clinical, and laboratory data were prospectively collected. The following stroke risk factors were identified: age, sex, hypertension (HTN), diabetes mellitus, dyslipidemia, current smoking, and a previous history of stroke or TIA. Baseline data, including the National Institutes of Health Stroke Scale (NIHSS) scores, were collected from all patients, and stroke subtypes were stratified according to TOAST criteria after performing complete diagnostic profiling. For the evaluation of cardioembolism, we assessed ECG monitoring results, echocardiography (routine transthoracic echocardiography and optional transesophageal echocardiography or cardiac CT), and cardiac enzymes. For UD or OE, we assessed angiography (MRA, CTA or conventional angiography), coagulation profiles, and autoimmune laboratory exam results. We assessed the neurological status at admission and on each hospital day using the NIHSS.

### Imaging studies

According to our stroke imaging protocol, the patients underwent emergency magnetic resonance imaging (MRI) at the emergency department immediately after hospital admission. The MRI protocol consisted of DWI, fluid-attenuated inversion recovery (FLAIR), gradient echo (GRE) imaging, and time-of-flight MR angiography (MRA) in sequence. Routine follow-up imaging (DWI and GRE) was performed 5 days after the first MRI. In addition, DWI and GRE were also performed if neurologic deterioration was observed. The images were analyzed by 2 independent neurologists (JTK and MSP) who were blinded to the clinical data. Discrepancies were resolved by consensus. Relevant arterial steno-occlusion was defined as symptomatic arterial steno-occlusion with moderate to severe stenosis (≥50% of luminal narrowing) or occlusion based on MRA results.

### Management

Aspirin was orally administered to the study patients immediately after the brain MRIs. The initial ‘loading’ dose was 300 mg for all patients, followed by maintenance doses of 100 mg daily. A combination of aspirin and other antiplatelet agents (clopidogrel 75 mg or cilostazol 100 mg) was administered to patients with severe steno-occlusion or a previous history of stroke or coronary artery diseases and to prior antiplatelet users at the discretion of the treating physician on the day following admission. For the patients whose mechanism of stroke was assessed as cardioembolism after a complete diagnostic work-up, anticoagulants were prescribed instead of aspirin, and these patients were excluded from the study.

### Measurement of aspirin responsiveness

The aspirin reaction unit (ARU) was assessed using the VerifyNow system (Accumetrics, Inc., San Diego, CA, USA). The acute ARU (aARU) was consistently measured after 3 hours of aspirin loading in the emergency department. An aARU value of ≥550 IU was defined as biological aspirin resistance (BAR), which is consistent with previous definitions [15,16]. The physicians were blinded to the aARU values.

### Outcomes

NILs were analyzed as early radiological outcomes. NILs were defined as lesions separate from index lesions and were not detected on the initial DWI. The characteristics of the NILs were analyzed, and the lesion locations and patterns of NILs were described. The patterns of NILs were classified as perforating artery infarcts (PAI), pial infarcts (PI), border-zone infarcts (BI), territorial infarcts (TI), and lacunar infarcts (LI) using modified definitions from previous studies [17]. Specifically, LI were defined as small (<2 cm diameter) lesions in subcortical areas, and PAI were defined as larger lesions in subcortical areas, such as a striatocapsular infarction. This definition is a modification of a previous definition. ‘END’ was also analyzed and defined as an increase in the NIHSS score by ≥1 point or the development of new neurological symptoms (i.e., a subcomponent of the scale that was previously scored 0 is subsequently scored ≥1 point) between admission and day 5.

### Statistical analysis

We compared the baseline characteristics between patients with BAR and those without BAR and between patients with NILs and those without NILs. The percentage, mean (±standard deviation, SD), or median (interquartile range, IQR) is reported, depending on the variable characteristics. Categorical variables were analyzed using the χ2-test and Fisher’s exact test, as appropriate. Continuous variables were analyzed using the independent samples t-test or the Mann–Whitney U-test, as appropriate. Multiple logistic regression analysis was used to evaluate the independent factors associated with each outcome, which were adjusted by age, initial NIHSS score, HTN, and variables with p<0.2 in univariate analysis. Furthermore, to evaluate BAR as a predictor of NILs on follow-up DWI, the BAR was adjusted by covariates (age, NIHSS score, and relevant steno-occlusion). In addition, the subgroup analyses included age, sex, NIHSS strata (4 points), TOAST classifications, arterial steno-occlusion on angiography at baseline, mono or dual therapy after the aARU, prior use or non-use of antiplatelet agents, and the presence or absence of dyslipidemia. Odds ratios (ORs) and 95% confidence intervals (CIs) were calculated. The statistical significance of the interactions between BAR and the included factors of the subgroup analysis was evaluated using multivariable logistic regression analysis. A p value of <0.05 was considered to be statistically significant. All statistical analyses were performed using SPSS for Windows, version 17 (SPSS Inc., Chicago, IL, USA).

## Results

### General characteristics

A total of 1,027 patients with acute ischemic stroke were screened within 24 hours of symptom onset during the study period. Of these patients, 620 were excluded: 284 due to high-risk cardioembolism (such as atrial fibrillation), 172 due to thrombolysis or endovascular treatments, 52 due to malignant stroke, 39 due to other etiologies in the TOAST classification, 32 due to a lack of DWI lesions, 25 due to low platelet counts (<90,000/IU) or coagulopathies, 9 due to the lack of informed consent, and 7 due to aspirin-induced asthma or aspirin allergies. After providing informed consent, 40 additional patients were excluded: 20 due to early loss to follow-up, 7 due to detection of other etiologies (such as arterial dissection), and 13 due to newly detected atrial fibrillation during admission. Ultimately, 367 patients (mean age, 65.9±11.38 years) were analyzed in this study. BAR in aARU was detected in 60 patients (16.3%). The general characteristics of the patients are shown in Table 1. There were no significant differences in age, sex, risk factors, or initial NIHSS scores between the 2 groups. However, large artery atherosclerosis (LAA) in the TOAST classification and relevant arterial occlusion were less frequently observed in the patients with BAR than in those without BAR (LAA, 58.3% vs 71.3%, p = 0.074; relevant arterial occlusion, 16.3% vs 26.1%, p = 0.087). The frequency of prior use of antiplatelet agents was not significantly different between the 2 groups (22.1% vs 25.0%, p = 0.616).

**Table 1:** General characteristics of the patients.

	ARU<550 (N = 307)	ARU≥550 (N = 60)	*P*
Age (mean, SD)	65.8±11.5	66.6±10.8	0.620
Male (n, %)	186 (60.6)	39 (65.0)	0.564
NIHSS (med, IQR)	2.0 (2.0)	2.0 (3.0)	0.950
Risk factors (n, %)			
Hypertension	181 (59.0)	38 (63.3)	0.567
Diabetes	102 (33.2)	14 (23.3)	0.171
Dyslipidemia	53 (17.3)	10 (16.7)	>0.999
Smoking	70 (22.8)	16 (26.7)	0.509
Previous stroke	54 (17.6)	11 (18.3)	0.855
Prior antiplatelets use			
Aspirin	68 (22.1)	15 (25.0)	0.616
Others	38 (12.4)	6 (10.0)	0.828
TOAST classifications			0.074
LAA	219 (71.3)	35 (58.3)	
SVO	33 (10.7)	10 (16.7)	
Undetermined	55 (17.9)	15 (25.0)	
Steno-occlusion (n, %)			0.087
Stenosis	90 (29.3)	15 (25.0)	
Occlusion	80 (26.1)	11 (18.3)	
Dual therapy (n, %)	78 (25.4)	21 (35.0)	0.152

National Institutes of Health Stroke Scale, NIHSS; TOAST, the Trial of Org 10172 in Acute Stroke Treatment; LAA, Large artery atherosclerosis; SVO, small vessel occlusion.

### NILs on follow-up DWI

The general characteristics of patients with and without NILs and END are shown in Table 2 and S1 Table. Eighty-one patients (22.1%) had NILs, and 149 patients (40.6%) had enlarged lesions on follow-up DWI. There were no significant differences in sex, risk factors, or initial NIHSS scores between patients with NILs and those without NILs. However, patients with NILs were older than those without, and relevant arterial steno-occlusion was more frequently observed in the patients with NILs than in those without (p<0.001). The frequency of dual antiplatelet use was significantly different between the 2 groups (p = 0.048).

**Table 2:** Comparisons of patients with and without new ischemic lesions (NILs).

	NILs (N = 81)	No NILs (N = 286)	*p*
Age (mean, SD)	68.5±10.9	65.2±11.4	0.022
Male (n, %)	47 (58.0)	178 (62.2)	0.520
NIHSS (med, IQR)	2.0 (3.50)	2.0 (2.0)	0.276
Risk factors (n, %)			
Hypertension	53 (65.4)	166 (58.0)	0.250
Diabetes	24 (29.6)	92 (32.2)	0.687
Dyslipidemia	19 (23.5)	44 (15.4)	0.097
Smoking	17 (21.0)	69 (24.1)	0.656
Previous stroke	14 (17.3)	51 (17.8)	>0.999
Prior antiplatelets use			
Aspirin	19 (23.8)	64 (22.4)	0.765
Others	8 (10.1)	36 (12.6)	0.697
TOAST classifications			0.128
LAA	54 (66.7)	200 (69.9)	
SVO	4 (4.9)	39 (13.6)	
Undetermined	23 (28.4)	47 (16.4)	
Steno-occlusion (n, %)			<0.001
Stenosis	29 (35.8)	76 (26.6)	
Occlusion	37 (45.7)	54 (18.9)	
ARU (mean, SD)	477.8±65.4	474.9±65.6	0.461
ARU ≥550 (n, %)	17 (21.0)	43 (15.0)	0.233
Dual therapy (n, %)	29 (35.8)	70 (24.5)	0.048
Clopidogrel	27 (33.3)	49 (17.1)	
Cilostazol	2 (2.5)	21 (7.3)	

Furthermore, BAR was independently associated with NILs on follow-up DWI (Model 1, OR 2.00, 95% CIs, 1.01–3.96, p = 0.047; Model 2, OR 2.04, 95% CIs, 1.03–4.07, p = 0.04) (Table 3). The characteristics of NILs are shown in S3 Table. NILs were frequently in the same territory as the index lesions (79%), pial infarcts (61.7%), and located within the cortex (59.3%) (S2 Table).

**Table 3:** Associations between various events and aspirin resistance (aARU ≥550).

	Model 1	*p*	Model 2	*p*
New ischemic lesions				
ARU ≥550	2.00 (1.01–3.96)	0.047	2.04 (1.03–4.07)	0.04
END				
ARU ≥550	0.49 (0.21–1.14)	0.099	0.46 (0.20–1.07)	0.07

Model 1: adjusted by age, NIHSS, and symptomatic steno-occlusion.

Model 2: adjusted by age, NIHSS, symptomatic steno-occlusion, and dual therapy.

END was observed in 76 patients (20.7%). BAR was less frequently observed in the patients with END than in those without END (9.2% vs 18.2%, p = 0.080); however, the difference was not statistically significant. In a sensitivity analysis, when END was defined as a 2 or 4 point increase in the NIHSS scores, BAR was less frequently observed in the patients with END than in those without END; however, these differences were not statistically significant (END 2; 11.7% in BAR vs 21.5% in no BAR, p = 0.11 and END 4; 11.7% in BAR vs 18.2% in no BAR, p = 0.26). The frequency of prior use of antiplatelet agents was not significantly different between the 2 groups. After adjusting for age, initial NIHSS scores, HTN, and variables with p<0.2 in univariate analysis (relevant arterial steno-occlusion, TOAST classifications, diabetes mellitus, and dual therapy), the independent factors associated with END were relevant arterial steno-occlusion and diabetes mellitus (S3 Table).

### Subgroup analysis for BAR

**Fig 1:**
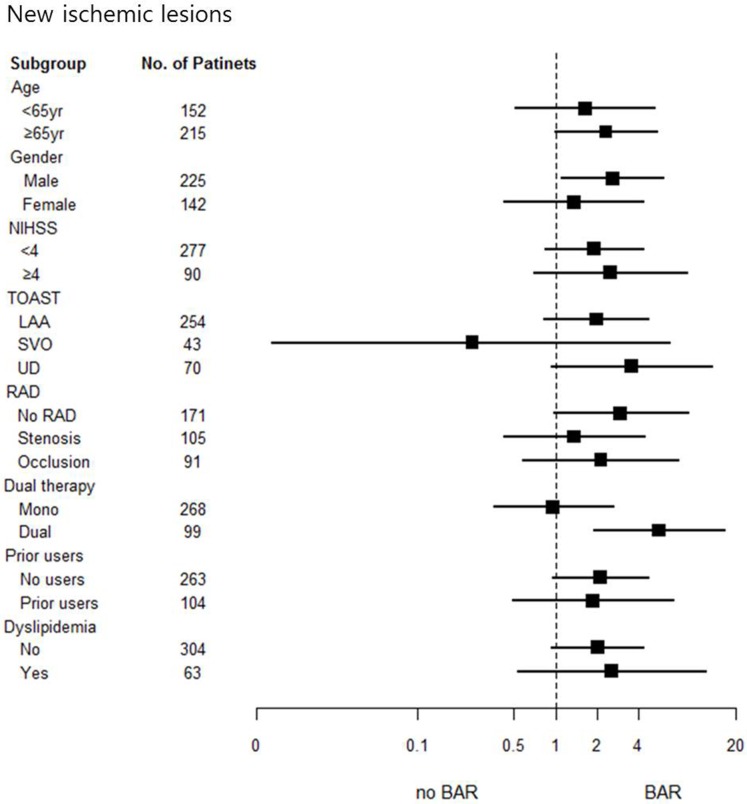
Subgroup analysis of NILs. A predefined secondary analysis for NILs on follow-up DWI showed that significant differences were present in the adjusted effect of BAR in patients who were male and had undergone dual therapy. Abbreviations: NILs, new ischemic lesions; RAD, relevant arterial diseases.

A predefined secondary analysis for NILs on follow-up DWI showed that significant differences were present in the adjusted effect of BAR in patients who were male and had undergone dual therapy. There were significant interaction effects between BAR and ‘mono or dual therapy’ (Fig. 1 and S4 Table). However, a secondary analysis of END showed no significant differences between the subgroups. Overall, no significant results were found, despite some interesting, albeit non-significant, signals in the adjusted effects of BAR on END (S4 Table).

## Discussion

Our study showed that BAR in aARU is independently associated with NILs on follow-up DWI in patients with acute ischemic stroke. The results of our study suggested that the aARU measurement might be helpful in predicting NILs on follow-up DWI, particularly in males and those who received dual therapy. This study was noteworthy because it used a prospective design to evaluate the clinical implications of the aARU in patients with acute ischemic stroke.

BAR may be explained by the following mechanisms: (1) reduced bioavailability (e.g., poor compliance), (2) altered binding to cyclooxygenase-1 (e.g., ibuprofen administration), (3) other sources of thromboxane 1 production, (4) alternative pathways of platelet activation, (5) increased platelet turnover, (6) genetic polymorphisms, (7) loss of antiplatelet effects with long-term administration of aspirin, and (8) non-atherothrombotic causes of cardiovascular events that were not expected to respond to antiplatelet agents [18]. However, issues such as poor compliance, interactions with concomitant medications, and non-atherothrombotic etiologies, would be expected to have minimal or no effects on our results because all patients in the study were inpatients and were strictly monitored through our prospective study protocols. For example, we excluded patients with other etiology in the TOAST classifications because of non-atherothrombotic causes of cardiovascular events that were not expected to respond to antiplatelet agents and those reporting chronic non-steroidal anti-inflammatory drug use because of altered binding to cyclooxygenase-1. Therefore, we presumed that the mechanisms of aspirin resistance in these patients might be the following: increased platelet turnover due to acute ischemic stroke, alternative pathways of platelet activation, or genetic polymorphism.

Jeon *et al*. have shown that some patients develop BAR and earlier recurrent ischemic lesions (ERILs) after aspirin loading [9]; however, because the study was retrospective in nature and small in size, the results should be interpreted cautiously. Although our study was a single center study, it was prospective in nature and had a larger sample size. Therefore, the results of our study and previous studies suggest that BAR in the acute stage could increase the risk of NILs on follow-up DWI in patients with acute ischemic stroke. ERILs that are representative of NILs on follow-up DWI could be a surrogate marker of subsequent stroke [19–21]. However, because our study did not directly investigate the associations between aspirin resistance and subsequent stroke, further study is warranted.

It has been shown that dual therapy may be more effective in protecting against the development of NILs than monotherapy in previous studies [22, 23]. However, the follow-up DWI in the subgroup treated with dual therapy demonstrated that NILs were more frequently observed in the patients with BAR than in those without. Our study protocol for dual therapy involved adding clopidogrel 75 mg or cilostazol 100 mg on the day after aspirin loading. Because clopidogrel usually requires 5 to 7 days to exert therapeutic effects in the absence of a loading dose, the addition of clopidogrel may not protect against NILs that develop (on follow-up DWI) within 5 days of administration in patients with BAR. Cilostazol 100 mg is also a subtherapeutic dose that is used for stroke prevention. Therefore, our results indicate that among the patients treated with dual therapy, BAR could be more significantly related to NILs on follow-up DWI than no BAR. In addition, because dual therapy was mainly considered in patients with symptomatic steno-occlusion, the risk of NILs may be higher in patients treated with dual therapy (S1 Table). Further studies are needed to determine how to manage patients with BAR after aspirin loading.

After aspirin loading, BAR was not associated with END in acute ischemic stroke. In our study, there were no significant relationships between BAR and END. Antiplatelet treatment did not appear to prevent END caused by lesion enlargement (such as swelling). In our study, END due to lesion enlargement accounted for >50% of all END cases. Therefore, the influence of BAR on END appeared to be masked by lesion enlargement. There were 16 cases of END due to NILs, which accounted for only 21% of all END cases, and 3 (18.8%) of these patients had BAR. Although our study did not support the hypothesis that the influence of BAR on END may be related to END due to NILs rather than lesion enlargement, NILs on follow-up DWI were independently associated with BAR. Bugnicourt *et al*. have indicated that aspirin resistance is predictive of END [10]; however, a small sample size and the presence of only 10 cases of END in the enrolled patients were limitations of that study. The present study suggests that BAR after aspirin loading could be associated with early radiological outcomes in patients with acute ischemic stroke. No studies have examined whether aspirin should be replaced with other antiplatelet agents (or dual therapy) when aspirin users develop NILs on follow-up DWI. In our study, the frequency of BAR after aspirin loading did not differ between prior users and never users of antiplatelet agents. Little biological evidence supports the premise that prior users of an antiplatelet agent who develop new ischemic stroke should receive another antiplatelet agent. However, Zheng *et al*. have reported that BAR is related to lesion volume and clinical severity in acute ischemic stroke patients who previously received aspirin therapy [15]. This result is inconsistent with our findings. In our study, all patients, including prior users of aspirin, were initially treated with a 300 mg loading dose of aspirin; however, there were no aspirin dose descriptions in the Zheng *et al*. study. In addition, because we excluded patients with malignant stroke from the study, the initial clinical severity of study subjects appeared to differ between the 2 studies. We measured the ARU after aspirin loading; thus, our study is more practical and appropriate for investigating the early clinical implications of BAR in acute ischemic stroke compared with some previous studies.

Our study has several limitations. We analyzed BAR in the aARU and the outcomes of patients with acute ischemic stroke during the first 5 days after aspirin loading. The ARU value may be influenced by the dose of aspirin and the timing of the ARU exam [24, 25]. Platelet hyper-reactivity despite aspirin administration was a frequent phenomenon in patients with acute ischemic stroke [25]. In a previous study, however, no difference was reported in the ARU values in subjects with exposure times from 1 h 50 m to 32 h after aspirin loading [26]. Therefore, the evaluation of aARU after aspirin 300 mg loading could be beneficial for detecting BAR in the acute stage of ischemic stroke. Furthermore, previous studies have demonstrated that early outcomes at 5 or 7 days might be representative of outcomes at 90 days [27, 28]. The lack of a consistent protocol for the use of antiplatelets was another limitation of the study. The addition of antiplatelets after aspirin loading was decided by the treating physician. However, there was no significant difference in the frequency of dual therapy between patients with BAR and those without. In addition, our study has the limitations that are inherent in single-center studies with relatively small sample sizes. Therefore, there was the lack of generalizability between genetically disparate Korean populations and other ethnic groups in other study.

In conclusion, after aspirin loading, BAR could be associated with NILs on follow-up DWI in patients with acute ischemic stroke. Therefore, further prospective studies with longer follow-up periods are warranted to evaluate the associations between the aARU in acute ischemic stroke and subsequent stroke.

## Supporting Information

S1 Text Brief description of the main study. (DOCX)

S1 Table Comparison of characteristics between patients with and without END. (DOCX)

S2 Table Characteristics of the new ischemic lesions (NILs). (DOCX)

S3 Table Factors associated with early neurological deterioration by multivariate logistic regression analysis. (DOCX)

S4 Table Subgroup analysis. (DOCX)
