# Supplementary material for: Aspirin Resistance in the Acute Stages of Acute Ischemic Stroke Is Associated with the Development of New Ischemic Lesions
Source: PLoS One. 2015 Apr 7;10(4):e0120743. doi: 10.1371/journal.pone.0120743 (PMC4388531; doi:10.1371/journal.pone.0120743)

**S1 Text.** Brief description of the main study.

**TITLE:** The Association of **RE**current **V**ascular **E**vents and Aspirin **R**esponsiveness in Patients with Acute Ischemic **S**trok**E** (REVERSE)

**PURPOSE**

To investigate the clinical implications of biological aspirin resistance (BAR) in acute ischemic stroke

**METHODS**

*Study design*

The REVERSE study was a prospective, single-center, observational study that was conducted between April 2012 and December 2013 with the aim of including 900 patients with acute ischemic stroke within 3 days of stroke onset.

This study was divided into three main studies (Figure). The first main study investigated the association between recurrent stroke and BAR after 5th days of aspirin administration to patients with acute ischemic stroke. The aspirin reaction unit (ARU) was assessed using the VerifyNow system (Accumetrics, Inc., San Diego, CA, USA). An ARU value ≥550 IU was defined as BAR. The physicians were blind to the ARU values. The primary outcome was recurrent stroke within 1 year.

The second main study investigated the early clinical implications of acute ARU (aARU) measurements in patients with acute ischemic stroke. This study included patients during the first 24 hours of symptom onset. The aARU was measured after 3 hours of aspirin loading in the emergency department. The primary outcome was early recurrent stroke within 3 months.

The third study investigated the clinical implications of difference between aARU and ARU at 5^th^ day of aspirin administration in acute ischemic stroke. Because of controversies of ARU measurements in acute ischemic stroke, we had to consider the third study in the middle of study.

Additionally, to investigate the acute clinical implications of ARU measurements, we assessed the clinical and radiological outcomes during the very early stage of ischemic stroke, such as early neurological deterioration (END) and new ischemic lesions (NIL) on diffusion-weighted imaging (DWI).

*Inclusion criteria*

- Part 1 study.

1. Within 3 days of stroke onset
2. Positive lesions on DWI
3. No potential risk of cardioembolism

- Part 2 study
  1. Within 24 hours of stroke onset (This inclusion criteria was changed into patients within 3days of stroke onset since July. 2012 in order to fast enrollments)
  2. Positive lesions on DWI
  3. No potential risk of cardioembolism
- Part 3 study
  1. Measurements of both aARU and ARU at 5^th^ day of aspirin administration
  2. Same as part 1 study

*Exclusion criteria*

- Part 1 study
  1. Other etiology of the TOAST classification
  2. Malignant infarction
  3. Chronic use of non-steroid anti-inflammatory drugs (>3 days per week during the most recent 3 months)
  4. A history of recent hemorrhagic disorders within the most recent 4 weeks
  5. Coagulopathies
  6. Thrombocytopenia (platelet counts >90,000/µl)
  7. Low hematocrit (<29%)
  8. Chronic liver diseases
  9. Chronic renal diseases
- Part 2 study
  1. Same as part 1 study
  2. Reperfusion therapy, such as intravenous tissue plasminogen activator administration
- Part 3 study
  1. Same as part 2 study

*Statistical analysis*

We compared the baseline characteristics between patients with BAR and patients without BAR. Kaplan-Meier curves were used to estimate survival free of primary outcomes in the part 1 and 2 studies. Cox-Hazard regression analysis was also used to evaluate the independent factors associated with the primary outcomes, which was adjusted by variables with p<0.2 in an univariate analysis (model 1) and those with clinical significances (model 2). Hazard ratios (HRs) and 95% confidence intervals (CIs) were calculated.

Part 3 study compared the values of aARU tests to those of ARU tests at 5^th^ day by paired *T* test. To evaluate clinical implications of ‘delta ARU (numerical differences between the values of aARU and ARU at 5^th^ day)’, equal sized groups of ‘delta ARU’ were adjusted for covariates to investigate the association with clinical outcomes. In addition, Kaplan-Meier curves were used to estimate survival free of primary outcomes. Cox-Hazard regression analysis was also used to evaluate the independent factors associated with the primary outcomes, which was adjusted by variables with p<0.2 in an univariate analysis (model 1) and those with clinical significances (model 2). Hazard ratios (HRs) and 95% confidence intervals (CIs) were calculated.

Figure. Schematic diagram of the study protocol.


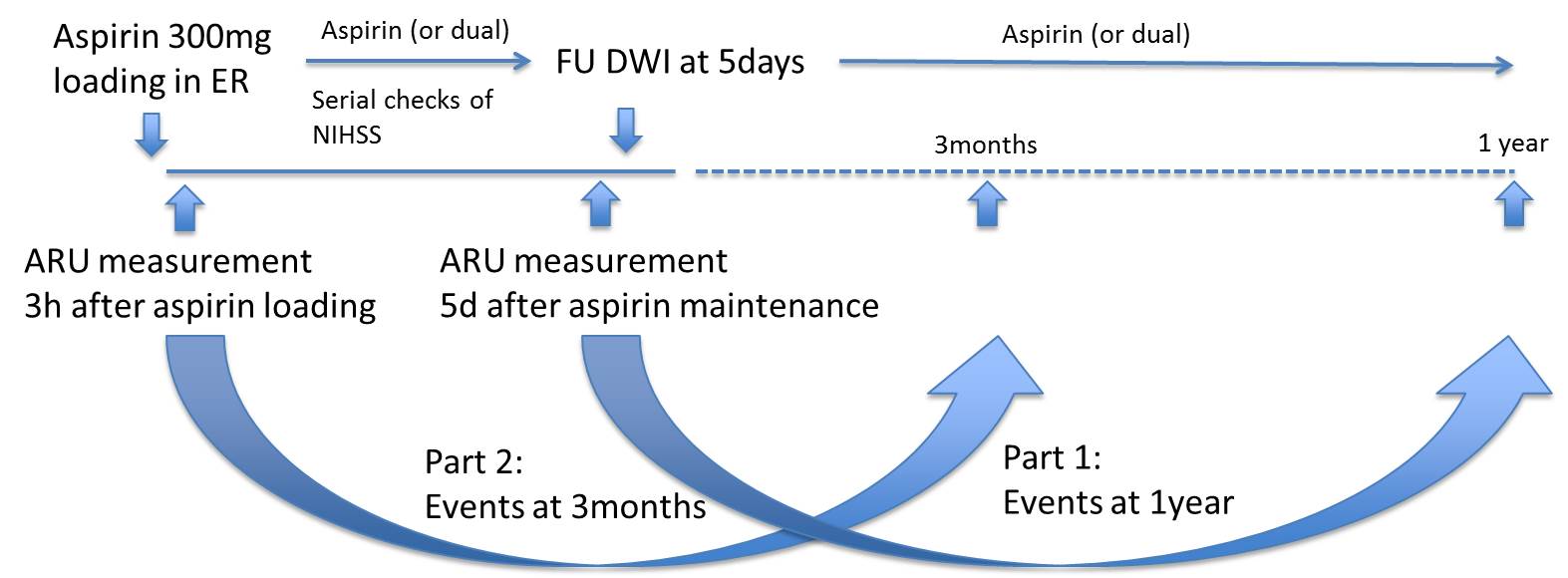

Supplement: S1 Text — (DOCX) [file pone.0120743.s001.docx]
