# Supplementary material for: Aspirin Resistance in the Acute Stages of Acute Ischemic Stroke Is Associated with the Development of New Ischemic Lesions
Source: PLoS One. 2015 Apr 7;10(4):e0120743. doi: 10.1371/journal.pone.0120743 (PMC4388531; doi:10.1371/journal.pone.0120743)
Supplement: S1 Table — (DOCX) [file pone.0120743.s002.docx]

S1 Table. Comparisons of characteristics between patients with and without END.

|  | END  (n=76) | No END  (n=291) | *P* |
| --- | --- | --- | --- |
| Age (mean, SD) | 66.4±11.7 | 65.8±11.3 | 0.666 |
| Male (n, %) | 43 (56.6) | 182 (62.5) | 0.357 |
| NIHSS (med, IQR) | 3.0 (3.75) | 2.0 (2.0) | 0.042 |
| Risk factors (n, %) |  |  |  |
| Hypertension | 50 (65.8) | 169 (58.1) | 0.240 |
| Diabetes | 38 (50.0) | 78 (26.8) | <0.001 |
| Dyslipidemia | 12 (15.8) | 51 (17.5) | 0.865 |
| Smoking | 16 (21.1) | 70 (24.1) | 0.650 |
| Previous stroke | 13 (17.1) | 52 (17.9) | >0.999 |
| Prior antiplatelets use |  |  |  |
| Aspirin | 15 (19.7) | 68 (23.4) | 0.542 |
| Others | 7 (9.2) | 37 (12.7) | 0.552 |
| TOAST classifications |  |  | 0.150 |
| LAA | 60 (78.9) | 194 (66.7) |  |
| SVO | 3 (3.9) | 40 (13.7) |  |
| Undetermined | 13 (17.1) | 57 (19.6) |  |
| Steno-occlusion (n, %) | 50 (65.8) | 146 (50.2) | 0.020 |
| Stenosis | 20 (26.3) | 85 (29.2) | <0.001 |
| Occlusion | 30 (39.5) | 61 (21.0) |  |
| Dual therapy (n, %) | 27 (35.5) | 72 (24.7) | 0.081 |
| ARU≥550 (n, %) | 7 (9.2) | 53 (18.2) | 0.080 |
| ARU values (mean, SD) | 472.7±60.8 | 476.2±70.5 | 0.691 |

END, early neurological deterioration, LAA; large artery atherosclerosis, SVO; small vessel occlusion, ARU, aspirin reaction unit.
