# Supplementary material for: Aspirin Resistance in the Acute Stages of Acute Ischemic Stroke Is Associated with the Development of New Ischemic Lesions
Source: PLoS One. 2015 Apr 7;10(4):e0120743. doi: 10.1371/journal.pone.0120743 (PMC4388531; doi:10.1371/journal.pone.0120743)
Supplement: S2 Table — (DOCX) [file pone.0120743.s003.docx]

S2 Table. Characteristics of the new ischemic lesions NILs.

|  | NILs (n=81) |
| --- | --- |
| Territory  Same territory  Different territory | 64 (79.0)  17 (21.0) |
| Patterns of NILs  PAI  PI  BI  LI  TI | 6 (7.4)  50 (61.7)  8 (9.9)  10 (12.3)  7 (8.6) |
| Locations of NILs  Cortex  Basal ganglia  Brainstem  Borderzone  Cerebellum | 48 (59.3)  11 (13.5)  8 (9.9)  8 (9.9)  6 (7.4) |

PAI; Perforating artery infarct, PI; pial infarct, BI; borderzone infarct, LI; lacunar infarct, TI; territorial infarct, NILs; new ischemic lesions.
