# Supplementary material for: Aspirin Resistance in the Acute Stages of Acute Ischemic Stroke Is Associated with the Development of New Ischemic Lesions
Source: PLoS One. 2015 Apr 7;10(4):e0120743. doi: 10.1371/journal.pone.0120743 (PMC4388531; doi:10.1371/journal.pone.0120743)
Supplement: S3 Table — (DOCX) [file pone.0120743.s004.docx]

S3 Table. Factors associated with early neurological deterioration by multivariate logistic regression analysis.

|  | Adjusted OR for END | *p* |
| --- | --- | --- |
| Age | 0.998 (0.973-1.023) | 0.854 |
| Male | NA |  |
| NIHSS | 1.043 (0.956-1.138) | 0.344 |
| ARU≥550 | 0.487 (0.200-1.190) | 0.115 |
| Dual therapy | 1.479 (0.776-2.818) | 0.234 |
| Steno-occlusion |  | 0.040 |
| Stenosis | 0.831 (0.387-1.787) | 0.636 |
| Occlusion | 1.932 (0.957-3.901) | 0.066 |
| TOAST |  | 0.195 |
| LAA | 1.226 (0.598-2.515) | 0.578 |
| SVO | 0.38 (0.096-1.493) | 0.165 |
| Undetermined | Ref |  |
| HTN | 0.951 (0.522-1.733) | 0.869 |
| DM | 2.913 (1.645-5.161) | <0.001 |

END, early neurological deterioration, ARU, aspirin reaction unit; HTN, hypertension; DM, diabetes mellitus.

END was adjusted by age, NIHSS, ARU≥550, HTN, and variables with *P*<0.2 (steno-occlusion, TOAST, DM, and dual therapy)
