# Supplementary material for: Aspirin Resistance in the Acute Stages of Acute Ischemic Stroke Is Associated with the Development of New Ischemic Lesions
Source: PLoS One. 2015 Apr 7;10(4):e0120743. doi: 10.1371/journal.pone.0120743 (PMC4388531; doi:10.1371/journal.pone.0120743)
Supplement: S4 Table — (DOCX) [file pone.0120743.s005.docx]

S4 Table. Subgroup analysis

|  | Numbers | OR for END  (95% CI) | p-value | P-value by interaction | OR for NILs  (95% CI) | p-value | P-value by interaction |
| --- | --- | --- | --- | --- | --- | --- | --- |
| Age |  |  |  | 0.807 |  |  | 0.634 |
| Age<65yr | 152 | 0.52 (0.14-1.91) | 0.324 |  | 1.63 (0.51-5.24) | 0.410 |  |
| Age≥65yr | 215 | 0.42 (0.14-1.28) | 0.127 |  | 2.31 (0.99-5.41) | 0.053 |  |
| Gender |  |  |  | 0.829 |  |  | 0.379 |
| Male | 225 | 0.43 (0.14-1.30) | 0.133 |  | 2.57 (1.10-6.03) | 0.030 |  |
| Female | 142 | 0.52 (0.14-1.93) | 0.324 |  | 1.36 (0.42-4.33) | 0.607 |  |
| NIHSS |  |  |  | 0.590 |  |  | 0.725 |
| <4 | 277 | 0.38 (0.13-1.15) | 0.086 |  | 1.90 (0.83-4.32) | 0.127 |  |
| ≥4 | 90 | 0.62 (0.16-2.44) | 0.492 |  | 2.49 (0.69-8.98) | 0.162 |  |
| TOAST |  |  |  | 0.834 |  |  | 0.338 |
| LAA | 254 | 0.65 (0.25-1.65) | 0.361 |  | 1.97 (0.83-4.70) | 0.125 |  |
| SVO | 43 | 0.44 (0.02-10.58) | 0.615 |  | 0.25 (0.01-6.73) | 0.410 |  |
| UD | 70 | 0.35 (0.06-2.22) | 0.266 |  | 3.53 (0.92-13.54) | 0.066 |  |
| RAD |  |  |  | 0.476 |  |  | 0.641 |
| No RAD | 171 | 0.71 (0.24-2.14) | 0.541 |  | 2.96 (0.97-9.01) | 0.057 |  |
| Stenosis | 105 | 0.11 (0.01-2.03) | 0.137 |  | 1.36 (0.42-4.39) | 0.611 |  |
| Occlusion | 91 | 0.74 (0.18-3.02) | 0.678 |  | 2.13 (0.58-7.77) | 0.583 |  |
| Dual therapy |  |  |  | 0.777 |  |  | 0.020 |
| Mono | 268 | 0.51 (0.17-1.52) | 0.227 |  | 0.96 (0.36-2.61) | 0.942 |  |
| Dual | 99 | 0.40 (0.10-1.52) | 0.176 |  | 5.60 (1.88-16.72) | 0.002 |  |
| Prior users |  |  |  | 0.760 |  |  | 0.864 |
| No users | 263 | 0.42 (0.15-1.14) | 0.088 |  | 2.11 (0.95-4.69) | 0.066 |  |
| Prior users | 104 | 0.56 (0.11-2.77) | 0.477 |  | 1.85 (0.49-7.01) | 0.368 |  |
| Dyslipidemia |  |  |  | 0.901 |  |  | 0.793 |
| No | 304 | 0.47 (0.19-1.18) | 0.107 |  | 2.01 (0.93-4.33) | 0.076 |  |
| Yes | 63 | 0.40 (0.04-3.67) | 0.420 |  | 2.54 (0.53-12.25) | 0.247 |  |

Adjusted by age, NIHSS scores, symptomatic steno-occlusion, and dual therapy.

END, early neurological deterioration; NILs, new ischemic lesions; RAD, relevant arterial diseases.
